# Living with a frozen shoulder – a phenomenological inquiry

**DOI:** 10.1186/s12891-022-05251-7

**Published:** 2022-04-04

**Authors:** Suellen Anne Lyne, Fiona Mary Goldblatt, Ernst Michael Shanahan

**Affiliations:** 1 Rheumatology Department, Level 2, Flinders Medical Centre, Flinders Drive, 5042 Adelaide, South Australia; 2 College of Medicine and Public Health, Flinders University, Adelaide, South Australia

**Keywords:** Adhesive capsulitis, Pain, Disability

## Abstract

**Background:**

Frozen shoulder (adhesive capsulitis) is an inflammatory condition affecting the capsule of the glenohumeral joint. It is characterised by a painful restricted range of passive and active movement in all planes of motion. The impact of frozen shoulder on affected individuals remains poorly characterised. In this study we sought to better understand the lived experience of people suffering from frozen shoulder to characterise the physical, psychological and socioeconomic impact of the condition.

**Methods:**

A qualitative study using a phenomenological approach was undertaken. Purposeful sampling was used to identify individuals for interview. Semi-structured interviews were performed and continued until saturation was achieved. A biopsychosocial framework was used during the analysis in order to generate themes which best described the phenomenon and reflected the lived experience of individuals’ suffering from this condition.

**Results:**

Ten interviews were conducted, and five main themes emerged including; the severity of the pain experience, a loss of independence, an altered sense of self, the significant psychological impact, and the variable experience with healthcare providers.

**Conclusions:**

These findings offer an insight into the lived experience of individuals with frozen shoulder, both on a personal and sociocultural level. The pain endured has profound impacts on physical and mental health, with loss of function resulting in a narrative reconstruction and altered sense of self. Our findings illustrate that frozen shoulder is much more than a benign self-limiting musculoskeletal condition and should be managed accordingly.

**Trial registration:**

ANZCTR 12620000677909 Registered 28/04/2020 https://www.anzctr.org.au/Trial/Registration/TrialReview.aspx?id=379719&isReview=true

**Supplementary Information:**

The online version contains supplementary material available at 10.1186/s12891-022-05251-7.

## Key points

- Frozen shoulder is often poorly diagnosed and inadequately managed, and this study adds important insights on the lived experience of individuals suffering from frozen shoulder.
- The severity and pervasiveness of the pain endured, a loss of independence and patients’ altered sense of self are profound representations of living with a frozen shoulder.
- This study challenges us to reconsider whether our current treatment targets for frozen shoulder are appropriate.
- An emphasis on early and effective pain management and on managing the psychological sequelae of the disease emerge from this study as key treatment targets.

## Background

Frozen shoulder is a common but often under-recognised condition. It is an inflammatory and fibrosing condition affecting the glenohumeral joint capsule, characterised by shoulder pain and stiffness with significant resultant disability [1, 2]. The aetiology is unknown, however a number of risk factors have been identified including diabetes and shoulder trauma [1, 3]. Frozen shoulder is considered primary if it occurs spontaneously, or secondary if there is an antecedent event such as trauma. The combined prevalence is estimated between 3 and 5% in the general population, but rates as high as 20% are reported in people with diabetes [4, 5]. Peak age of onset is 40–60 years and women are affected slightly more often than men [6, 7]. Given frozen shoulder typically affects those of working age, there are resultant economic impacts, both on a personal and societal level [8].

Frozen shoulder is predominantly a clinical diagnosis. Restriction of movement occurs in all planes of motion, both passive and active, and there is insufficient joint degeneration to account for the restricted movement [9]. The pathognomonic feature is almost complete loss of external rotation [7]. Additional investigations are usually non-contributory but can be useful in ruling out other pathologies. The natural history of frozen shoulder remains poorly understood [10], but the most commonly accepted description of disease progression is defined by three overlapping clinical phases. Phase one manifests as severe shoulder pain, typically worse at night, with concurrent progressive loss of motion. This phase can last 2–9 months and is known as the *painful phase*. Phase two, the *frozen phase*, lasts 4–12 months and is characterised by gradual reduction in pain, but persistent and considerable restriction in movement. Phase three, termed the resolution or *thawing phase,* and can last 12–36 months [3]. Few effective treatments are available which significantly alter the natural history of disease [2]. The extent of recovery is variable, with some reporting persistent pain and residual limitation of movement. In one large case series 35% of people had mild to moderate and 6% had severe symptoms, at a mean follow up of 4.4 years [11]. Recurrence is uncommon, although the contralateral shoulder can become affected in 6–17% of patients within the first 5 years [11]. In current practice the management of frozen shoulder has been primarily undertaken by orthopaedic surgeons and physical therapists who emphasise biomechanics and restoration of range of motion. This approach has tended to shape our understanding of the condition and influence treatment targets.

The clinical picture of frozen shoulder is well described but the impact on individual suffering is poorly characterised. Some people describe difficulties with basic activities of daily living, such as showering, dressing and cooking [8]. The pain is reported to interfere with sleep, which further intensifies the pain and impacts one’s ability to engage with domestic, social and occupational activities [12, 13]. What remains poorly described is the experience of protracted and debilitating shoulder pain which has the potential for profound physical, psychological and socioeconomic consequences [14]. A recent systematic review of patients’ experiences with shoulder disorders in general concluded that patients contend with considerable disruption to their lives, impacting sleep, cognitive function and emotional wellbeing [12]. However, there is very limited data reporting the impact of frozen shoulder. One paper focused on patients’ experiences with conventional care pathways [8] and another examined patients’ experience of a specific treatment, Bowen’s technique [15]. To our knowledge, there have been no papers describing a holistic exploration of the lived experience of frozen shoulder.

Despite its prevalence, treatment outcomes for frozen shoulder continue to be modest [2]. Given this reality, it is important to ask why our treatments appear to be missing the mark. Is it possible our therapeutic targets are not the most appropriate for this poorly understood condition? Little is reported about the experience of individuals suffering from frozen shoulder, so in this paper we set out to better understand the lived experience. We believe this work to be important in helping to better manage this common medical condition. By improving understanding of the impact on the individual, we aim to increase practitioner awareness of the disease and its severity, facilitate earlier diagnosis and better design therapies which improve outcomes that are important to patients [16].

## Methods

A qualitative study using a phenomenological approach was employed to explore the lived experience of a group of individuals suffering from frozen shoulder. Participants were identified from a group of patients with a recent diagnosis of frozen shoulder based on assessment by a specialist Rheumatologist. Inclusion criteria included male and female participants, aged over 18 years. There were no exclusion criteria. Patients were referred to the Southern Adelaide Local Health Network (SALHN) rheumatology outpatient clinic from community and hospital settings, including general practice, physiotherapists and specialty services, with the exception of one participant who was not previously known to the service. Purposive sampling was used to select individuals for interview. Patients who reported significant psychosocial impact from their frozen shoulder were invited to participate.

Interviews took place either in person at the rheumatology outpatient clinic or via audio and video telehealth, between June and August 2020. In-depth, semi-structured interviews were conducted using an interview guide (Supplementary File 1) Following an introduction to orientate participants, the interviewer asked the interviewee to describe the impact of the frozen shoulder on their life. Questions were “directed to the participants’ experiences, feelings, beliefs and convictions about the theme in question” [17]. An iterative approach was used for the interviews, meaning that knowledge acquired in each interview helped guide questioning for interviews of subsequent participants and enabled identification of emergent themes [18]. The audio-recorded interviews were transcribed and data de-identified. Each transcript was given to the respective interviewee to read, and an opportunity provided to amend or add any material they felt relevant. Interviews were conducted by a single researcher, SL, who had no involvement in the participants’ medical care prior to study commencement, minimising risk of bias. SL is a rheumatologist who had professional knowledge of the condition and was trained in qualitative interview techniques. Interviewing was continued until saturation was achieved, that is until interviewees introduced no new perspectives, no new themes emerged, and no further coding was possible [19, 20]. The SALHN Human Research Ethics Committee approved the study (OFR number: 81.20, ANZCTR 12620000677909).

A phenomenological approach was deemed the most appropriate enquiry method because the research sought to understand the participants’ conscious experience; their judgments, perceptions and emotions [21–23]. The philosophy of Husserl and methods described by Colaizzi were applied to analyse the interviews [21, 24]. This involves reading the interviews in their entirety, repeatedly if necessary, in order to become familiar with the data set and develop a holistic sense of the interview, the “gestalt” [25]. The researchers attempt to bracket out their own prejudices and presuppositions to avoid prejudging the data and therefore allow true realisation of the essence of the experience in order to “enter the unique world of the informant/participant” [26, 27]. Coding was conducted using NVivo 12 Qualitative Data Analysis Software [28]. Coding was performed by the three authoring researchers and all interviews were at least dual coded. The authors are rheumatologists with experience in qualitative research. Codes were collated and sorted, and units of meaning delineated, taking into account the literal content, the number of times the unit of meaning arose, and how the meaning was delivered. Themes were derived from the data rather than being identified in advance. Themes emerged as units of meaning were clustered, bringing together recurrent experience and its variant manifestations, in order to get to the essence of the phenomenon and elucidate the “lived experience” [29, 30].

## Results

Of the 16 individuals invited, ten agreed to participate (Eight female and two male, age range 32–72). No participants were excluded, and all provided written informed consent. Four did not respond to our invitation and two others expressed interest but we not formally recruited before saturation was achieved. Nine interviewees had recovered from their frozen shoulder, having been diagnosed within the preceding 2 years, while one was still in the resolution phase. Two participants had suffered two frozen shoulders, affecting each shoulder at different time points, whereas the remainder had only one arm affected. Data analysis resulted in identification of five main themes; the severity of the pain experience, a loss of independence, an altered sense of self, the significant psychological impact, and the variable experience with healthcare providers.

### The severity of the pain experience

The intractable nature and intensity of the pain dominated the participants’ experience of frozen shoulder. The pain was severe and the timing unpredictable, although most often precipitated by movement. Participants used vivid descriptors of pain, such as “horrible”, “excruciating”, “debilitating” and “unbearable” to illustrate their experience.

*“You see the Western movies [where] they forge the steel and … plunge it into the cold water … that’s what it feels like, hot, molten lava encapsulated by metal, dragging your shoulder down”*

The pain affected all facets of life, impacting work, sleep, personal hygiene, interpersonal relationships and independence. The prolonged duration and inescapable nature of the pain made it both debilitating and demoralising. It was consistently recognised that above all else, participants wanted the pain to go away. Pain was the priority and was considered far more significant than the loss of movement:

*“All I wanted was for the pain to go away”.*

Resolution of the pain had a major positive psychological impact on participants:

*“Once the pain was alleviated, then I could cope with everything else”.*

Improvement in pain enabled participants to engage more effectively in physical therapies and begin their recovery.

### A loss of Independence

The degree and impact of the disability and loss of independence was another prominent theme. There were implications both at home and in the workplace. Activities such as dressing, driving, shopping and personal hygiene were limited, or near impossible for some, occasionally with resultant loss of income. This was particularly evident for those whose dominant arm was affected.

*“It's hard to describe how much it limits you. You can't do anything”.*

Many participants felt that the severity of their illness and consequent disability was underappreciated by others due to the ‘invisible’ nature of frozen shoulder. Often a sense of stoicism was expressed as people tried to cope as though nothing was wrong, while simultaneously becoming frustrated by others’ inability to appreciate their incapacity. Many struggled to accept their disability, and were reluctant to rely on others:

*“I felt like I was abusing my friends … to ask them again or to rely on them again, that was stupid”.*

Meanwhile, others exhibited a greater sense of acceptance and self-kindness. There was an apparent sense of fear associated with loss of independence; fear of being judged, of being a burden, of resentment, and of loss of income. This fear was compounded by social isolation and a sense of hopelessness, particularly for those with limited social supports and inadequate access to services.

### An altered sense of self

The loss of independence, in combination with intractable pain, prompted an identity narrative reconstruction for some participants, resulting in an altered sense of self. In the context of the severe pain and physical limitation, individuals struggled to meet their own expectations, and also the expectations of those around them.

*“I was supposed to be the one that could cope and do everything”*

This resulted in a growing separation between their private sense of self and their public social identity, causing a paradigm shift in their self-concept [30–32]. Pain was a constant reminder of disability. Their disability prevented them for undertaking activities of daily living, as well as activities that brought joy, such as sport and playing with children or grandchildren. Inability to drive or catch public transport caused their world to shrink. Interrupted sleep caused fatigue and irritability, as well as an inability to function at full capacity at work. The fear of being a burden and lack of understanding from others resulted in social isolation:

*I didn't want anybody around me because I didn't want anybody to see me the way I was”*

Cumulatively, these factors challenged the sufferers’ perception of self and often resulted in social withdrawal. For some, this altered sense of self was enduring, and in others it was transient. Resilience and strong supportive social networks were recognised as important factors in preventing such a loss in sense of self.

### The psychological impact

Living with a frozen shoulder often had profound psychological effects including anxiety, irritability, depression and even the contemplation of suicide. The unrelenting pain, as well as interrupted sleep, were key determinants of mood:

*“When the world is crumbling and you’re in pain … it’s a dark time”*

Furthermore, patients often felt they weren’t believed about the severity of their pain, with a lack of empathy and understanding emanating from family, friends, colleagues and members of the medical profession. People reported feeling frustrated at their lost independence and lack of understanding from others. This frustration manifest itself as irritability.

*“people see me as more cantankerous than they’d ever been seen before.”*

Depression was frequently reported.

*“I’d had enough, I couldn’t handle it and my depression got really bad”*

Two participants had even contemplated suicide as a means of ending their pain.

*“had I woken up the next morning … still in the same sort of pain, then yes, I may not still be here”*

A lack of recognition of the individuals’ mental health issues by treating health professionals was reported, and may have contributed to the psychological impacts being poorly managed.

### The healthcare approach

Medical and allied health professionals often appeared to lack awareness of this condition. At times, this resulted in prolonged delays to diagnosis, inappropriate investigation and inadequate treatment.

*“I’d seen 3 or 4 GP’s up to that point and none of them, they’re all sending me off to have an ultrasound for bursitis and no one, and I’d even mentioned frozen shoulder and, and they were all like “No we don’t think it’s that” you know, and so I felt very disempowered from the medical profession and very not understood.”*

There were many differing interpretations by the health professionals of what “frozen shoulder” actually meant and why people get it. On reflection, participants became aware of this knowledge gap over time. An apparent lack of compassion and empathy was sometimes noted as members of the healthcare team trivialised the severity of symptoms and failed to acknowledge their impact.

*“[They] never ever asked me how things, how was I managing at home”*

The severity of the pain was also underappreciated by certain practitioners, as demonstrated by the lack of compassion when asked to perform exercises:

*“She grabbed my arm, and she used all her force to pull my arm all the way up, I screamed like you had no idea.”*

Participants expressed concerns about the overall knowledge and experience of many of their healthcare providers and suggested a need to improve this through further education. In contrast, participants described a sense of relief when a health practitioner was able to identify and confidently manage their frozen shoulder. The knowledge of being understood provided a sense of relief, and instilled patients with new hope.

*“I almost burst into tears. I can feel it now. I was like*

*Oh my God, someone understands me”*

The formation of a therapeutic relationship and established treatment targets was evidently important in the overall healing process.

## Discussion

To our knowledge, this is the first qualitative study to describe the phenomenon of living with a frozen shoulder. Jones et al. explored the perceptions of people living with frozen shoulder and their priorities for treatment [8] however, while this work emphasised the loss of function and unacceptable delays to treatment, there was little evaluation of the subjective experience and psychological impact of the pain.

The aetiology and pathophysiology of frozen shoulder remains poorly understood and is postulated by some to be an algoneurodystrophic process, on the same spectrum as chronic regional pain syndrome [33, 34]. It is difficult to delineate the role of tissue damage and nociceptive stimulation in perception of pain from frozen shoulder. According to Moseley, pain experience does not correlate directly with the state of the tissues and many factors across somatic, psychological and social domains influence pain modulation [35]. In the absence of a clear pathological process and inadequate direction from health practitioners, the sufferer’s consciousness is held accountable for their own condition [36, 37]. The complexity of musculoskeletal pain has been extensively studied and there is an ever-growing body of evidence which explores the modern concept of chronic pain [35, 38–40]. It has been consistently demonstrated that expectations and pre-existing personality traits influence one’s experience of pain. For example, catastrophising and health-related anxiety are associated with heightened pain experience, whereas resilience and strong social supports help ameliorate pain [40, 41]. Given the burden of psychological distress expressed by some of our participants, it is imperative that patient expectations, social isolation and health related anxiety be acknowledged and addressed early when managing frozen shoulder.

Physical therapy is considered by many as first line therapy for frozen shoulder despite multiple studies and a recent Cochrane review demonstrating little evidence to support physical therapy [34, 42–44]. Our work has highlighted that the patients’ priority is pain management, above all else. Patient-centred care involves listening to patients’ priorities [16] and empowering patients to effectively critique and provide feedback on the quality and appropriateness of healthcare services [45]. In our study, pain management clearly was prioritised by the patients over range of movement. By treating the pain effectively, participants said that they would be better able to cope psychologically with their incapacity and engage better with physical therapies.

Our data showed that being understood by healthcare professionals and others also played a therapeutic role. Participants reported feeling frustrated and disempowered by the lack of understanding and empathy offered by health professionals. This sentiment has been echoed by other qualitative work exploring chronic pain, even in specialist pain clinics, where it seems the clinician’s ability to truly understand the patients’ experience is “outside their lexicon of knowledge” [8, 14]. If a patient’s experience is misrepresented or trivialised by the health practitioner, this may propagate a negative self-image with resultant shame, distress and altered sense of self [46]. Such feelings promote resentment and helplessness and are counterproductive to the therapeutic relationship, while also increasing risk of anxiety and depression [38]. This in turn magnifies the individual’s experience of pain. Conversely, if a patient feels their struggle is understood, there are profound positive impacts. It validates the individual’s experience, which instils a sense of relief and hope, reaffirms their functioning as a social individual and diminishes their sense of spoiled identity [14]. There is a large body of evidence which supports the need for a strong therapeutic alliance when managing chronic musculoskeletal pain, based on a shared understanding of the symptomatology and treatment targets, and the management of frozen shoulder is no different [47, 48].

The findings in this research have allowed us to reconceptualise the treatment targets for frozen shoulder and a number of issues have been identified which may improve patient care. Firstly, a clearer focus on early and effective pain management is imperative, as is improved management of the psychological sequelae. These findings align with an increasing body of evidence that explores the impact of pain and pain-related behaviours on outcomes in frozen shoulder [49–51]. Treatment of chronic musculoskeletal pain is complex, requiring a multifaceted biopsychosocial approach. While targeting nociceptive signals with analgesia and neural blockade may be helpful, educating patients about the mechanisms of chronic musculoskeletal pain is also of great importance [52]. Enquiring about a patient’s coping strategies and acknowledging their struggle are simple measures to provide improved psychological support. In Australia, there is a paucity of services available for patients with short term incapacity, however, referral to a psychologist and advocating for access to community support services may be beneficial. Education is required to enable practitioners to better recognise and manage frozen shoulder. Equally, the language chosen by clinicians needs to be reconsidered in order to foster a constructive therapeutic alliance [53]. Given that frozen shoulder is so painful and slow to improve, early patient education is critical to ameliorate apprehension and help formulate realistic treatment targets [4]. We believe that addressing the above short fallings will reduce pain severity, lessen the sense of spoiled identity and help minimise rates of anxiety and depression.

There are a number of strengths to our study. The use of bracketing and dual coding has minimised potential bias. Employing phenomenology as the methodology in this study has allowed the voice of individuals’ experiences to be clear. Some limitations to this study also need to be considered. Participants’ experiences were historical, having recovered from their frozen shoulder some weeks or months prior and it is therefore possible that themes may have differed in participants with active disease. Recall bias must also be taken into consideration. Because of public health restriction in place at the time of our study, interviews were conducted in person, and via audio and video telehealth. Use of different communication platforms may have impacted rapport and limit translatability. Furthermore, this was a small study which included mostly patients from a single tertiary centre and limited geographical area.

This study has focused on the experience of living with a frozen shoulder. It has provided new insight into the severity of the pain and the subsequent impact on physical and mental health, with loss of function resulting in a narrative reconstruction and altered sense of self. Insights from this research align with recent studies that emphasise the importance of pain-perception and pain-related behaviours in patients with frozen shoulder. Increased practitioner awareness of patient experience helps facilitate earlier diagnosis and refocuses treatment approaches based on patient priorities. A clearer emphasis on early and effective pain management and on managing the psychological sequelae of the disease are two clear opportunities which emerge from this study.

## Supplementary Information

**Additional file 1.**

## Data Availability

De identified original interviews available from the corresponding author upon reasonable request.
