# Supplementary material for: Living with a frozen shoulder – a phenomenological inquiry
Source: BMC Musculoskelet Disord. 2022 Apr 4;23:318. doi: 10.1186/s12891-022-05251-7 (PMC8978403; doi:10.1186/s12891-022-05251-7)
Supplement: Supplementary file 1 — Additional file 1. [file 12891_2022_5251_MOESM1_ESM.docx]

**Living with a Frozen Shoulder - A Phenomenological Inquiry**

SA Lyne, FM Goldblatt, EM Shanahan

**Supplementary Material - Interview Guide**

1. I would like to hear about your experience with your frozen shoulder, I wonder if you could tell me about it.
2. What were some of the problems you had when living with your frozen shoulder?
3. What was the worst experience you had?
4. Can you please tell me about your experience with the pain from the frozen shoulder?
5. In what way did the frozen shoulder affect your ability to live your life?
6. Did the frozen shoulder impact on your relationships? If so, in what way?
7. Did the frozen shoulder impact on your work? If so, how?
8. Did the frozen shoulder affect your ability to sleep?
9. Did the frozen shoulder impact on your ability to look after yourself?
10. Did the frozen shoulder affect your physical health in any way?
11. Did the frozen shoulder affect your mental well-being? Mentioned severe depression and suicidal thoughts, but no crying
12. What was your experience of the treatment of the frozen shoulder?
13. Do you think your doctors and physiotherapists understood what you were experiencing?
14. Was there a financial impact on you as a result of the frozen shoulder?
